# Lung T cell response in COVID-19

**DOI:** 10.3389/fimmu.2023.1108716

**Published:** 2023-02-16

**Authors:** Mehrnoush Hadaddzadeh Shakiba, Ioanna Gemünd, Marc Beyer, Lorenzo Bonaguro

**Affiliations:** ^1^ Systems Medicine, Deutsches Zentrum für Neurodegenerative Erkrankungen (DZNE), Bonn, Germany; ^2^ Immunogenomics and Neurodegeneration, Deutsches Zentrum für Neurodegenerative Erkrankungen (DZNE), Bonn, Germany; ^3^ Genomics and Immunoregulation, Life and Medical Sciences (LIMES) Institute, University of Bonn, Bonn, Germany; ^4^ Department of Microbiology and Immunology, Peter Doherty Institute for Infection and Immunity, The University of Melbourne, Parkville, VIC, Australia; ^5^ PRECISE Platform for Single Cell Genomics and Epigenomics, Deutsches Zentrum für Neurodegenerative Erkrankungen (DZNE) and University of Bonn, Bonn, Germany

**Keywords:** T-cell, COVID-19, SARS-CoV-2, pathomechanism, lung

## Abstract

The COVID-19 pandemic has shown the potentially devastating impact of novel respiratory infections worldwide. Insightful data obtained in the last years have shed light on the pathophysiology of SARS-CoV-2 infection and the role of the inflammatory response in driving both the resolution of the disease and uncontrolled deleterious inflammatory status in severe cases. In this mini-review, we cover some important aspects of the role of T cells in COVID-19 with a special focus on the local response in the lung. We focus on the reported T cell phenotypes in mild, moderate, and severe COVID-19, focusing on lung inflammation and on both the protective and damaging roles of the T cell response, also highlighting the open questions in the field.

## Introduction

Coronavirus disease 2019 (COVID-19), the pathology caused by the recently emerged severe acute respiratory syndrome coronavirus 2 virus (SARS-CoV-2), has led to a global pandemic of severe respiratory disease with relatively high morbidity and mortality. As of January 2023, more than 6.7 million deaths were reported during several waves of virus variants with multiple mutations emerging over time, challenging immunological memory and vaccination strategies (WHO).

SARS-CoV-2 infection leads to a wide range of symptoms, ranging from an asymptomatic to mild/moderate infection up to severe disease requiring hospitalization and mechanical ventilation, often progressing to acute respiratory distress syndrome (ARDS).

The SARS-CoV-2 virus is part of the Conanaviridae family; structurally, its ~30 kb long positive-sense RNA genome encodes for a total of 29 proteins of which four have structural function (Spike (S), Envelope (E), Membrane (M), and Nucleocapsid (N) proteins) (1). From an immunological point of view, it is well-known that antibody responses against other coronaviruses are not well maintained, and reinfections are common within 12 months (2). Similarly, effective T cell responses against human coronaviruses are generated frequently but are of relatively low magnitude, and their longevity is uncertain, with low frequencies of antigen-specific T cells, especially in older people (3). Furthermore, mutations in several structural proteins, especially Spikes, are relatively common (4) and can bypass the existing immunological memory from both previous infections and vaccinations (5).

The respiratory tract is the main pathological site of SARS-CoV-2 infection, with the lung often being the major affected organ, especially in severe cases. For many severe COVID-19 patients who succumbed to the disease, the lung is histologically characterized by diffuse alveolar disease (DAD). DAD is the histological hallmark of acute ARDS and is characterized by edema, hyaline membranes and inflammation, usually followed by alveolar septal fibrosis (6). Nevertheless, some patients also show a distinct inflammatory milieu specific to COVID-19 (7), while others have an extrapulmonary manifestation of the disease caused by lung thrombosis (8). Despite being the location where the disease manifests, most clinical investigations of COVID-19 are based on blood sampling, lung tissue samples were derived from deceased patients, making it difficult to fully understand the spectrum of the disease (7, 9).

During the early phase of the COVID-19 pandemic, it became clear that the immune system also causes tissue damage due to uncontrolled inflammation, where the innate response, monocytes, and neutrophils, play a significant role (10). Understanding the immune response to this novel virus is extremely important to efficiently design therapies for severe cases beyond antiviral treatments such as dexamethasone, specifically aiming at this exacerbated immune reaction. The adaptive immune response plays a key role in viral clearance and the formation of immunological memory. In this mini-review, we focus on the role of T cells in the lung in COVID-19, summarizing recent work and providing a perspective for the successful treatment of COVID-19.

## Characterization and function of T cells in the human lung

**Figure 1:**
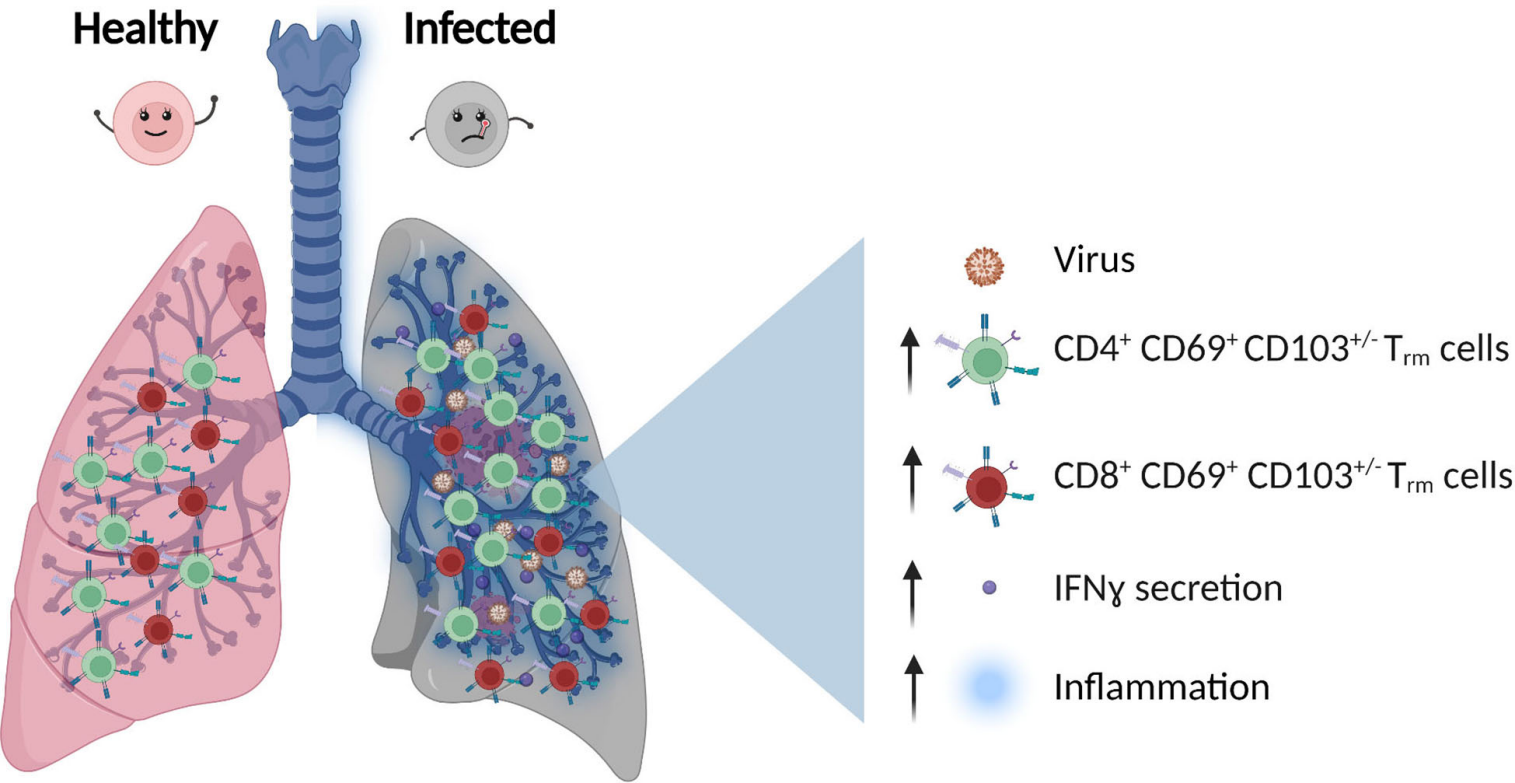
T Cells population in healthy vs infected lung. After viral infection, the number of CD4^+^ and CD8^+^ effector-memory, tissue-resident T cells (T_rm_), and inflammatory cytokines secretion such as IFNγ (Interferon gamma) increase.

As an organ at the interface with the environment, the human lung requires constant immune protection and surveillance while maintaining tissue homeostasis. To achieve this, the lung hosts highly heterogeneous populations of innate and adaptive immune cells, most of which are tissue-resident (11). Besides alveolar macrophages, T cells are the second most abundant immune cells residing in the lung (11), mostly with the phenotype of effector-memory, tissue-resident T cells (T_rm_, CD69^+^, CD103^+^/-, CD45RA, CCR7^-^). These cells provide protection against many pathogens (11), but can also promote immunopathology when a dysregulated interaction between immune cells and lung tissue is established (12, 13). Although most studies reporting on T_rm_ are based on murine experiments, mainly due to difficulties accessing human lung tissue, it is now established that also in human lungs, most of the T cells have a memory phenotype (CD4^+^ and CD8^+^) and are tissue-resident (14–17). Those cells are found across the entire respiratory system including the lung parenchyma, airways and even associated lymph nodes (18) (**Figure 1**).

T_rm_ cells originate from naïve T cells that have been primed in lymphoid tissues and then migrate to the lung after antigen exposure (19) giving rise to the most frequent T cell population in adult lungs (11). Considering the origin of lung tissue-resident T cells, the composition of this cell population varies according to the donor’s age and individual history. In the first few years of life, the structure of the T cell population in the lung changes from being dominated by circulating naïve T cells (CCR7^+^CD45RA^+^) and T_reg_ cells (CD4^+^CD25^+^FOXP3^+^) to antigen-experienced T_rm_ from late childhood into adult life persisting for many decades (19, 20). The question of how long this cell population lasts in elderly people where thymic contribution is almost absent and T cells show a more senescent and anergic phenotype is still not fully understood. Interestingly, several viral infections of the lung cause more severe disease in individuals with signs of immunosenescence (21) pointing toward a reduced capacity of these cells to respond to antigens or a rather general incapability to generate a new antigen-specific response from the naïve pool.

Lung T_rm_ cells accumulate and reside around the airway and have the potential to rapidly respond to both viral and bacterial reinfections by rapid secretion of IFNγ (11) (**Figure 1**). Furthermore, recent single-cell TCR (T cell receptor) sequencing showed that influenza-specific T_rm_ cells maintain a certain degree of receptor diversity, highlighting the potential cross-reactivity to multiple virus strains (22). Considering the importance of these cells in protection against reinfection, their expansion upon vaccination is of special interest. In an influenza model, intranasal administration of an attenuated influenza vaccine led to increased frequency of T_rm_ cells and better protection from infection compared to conventional parenteral injections (22), highlighting the benefit of vaccines that induce T cells responses acting directly at the site of pathogen entry.

Despite their crucial protective role, the pool of T_rm_ cells functionally also harbors potentially damaging effects. Recently, they have been indicated to directly contribute to psoriasis and mycosis fungoides, as well as asthma and allergic airway diseases (23). E.g. in a house dust mite mouse model, T_rm_ cells polarize towards an airway resistance-promoting Th2 (T helper 2) phenotype (11), while lung memory CD8^+^ T cells post influenza infection were shown to modulate an inflammatory response to common antigens independently from their TCR specificity (24).

## Role of human T cells in COVID-19 lung inflammation

### Peripheral T cell response to SARS-CoV-2

The adaptive immune system is crucial in controlling SARS-CoV-2 infection through the coordinated action of B cells (producing antibodies) and T cells with helper (CD4^+^) or killer (CD8^+^) functions (25). In the following paragraph, when phenotypic and functional characteristics are shared between CD4^+^ and CD8^+^ T cell compartments, we refer to T cells; otherwise, we define the T cell subset that is affected.

A key feature of severe COVID-19 is peripheral lymphopenia, which is reverted with disease resolution (26). This change in T cell numbers is accompanied by long-lasting phenotypic changes persisting in the remaining T cells ranging from activated to fully exhausted/dysfunctional phenotypes (27). Almost all COVID-19 patients develop a T cell response, which is more prominent in the CD4^+^ compartment than in the CD8^+^ compartment (28). The number of CD4^+^ T cell clones against SARS-CoV-2 is correlated with the abundance of the structural protein, with Spike [also the target of all available vaccines (29, 30)], M, and Nucleocapsid as the most prominent antigenic targets.

In COVID-19, as in many other viral infections, CD4^+^ T cells are crucial for providing help to CD8^+^ T and B cells (25–27). Further, the absence of a CD4^+^ T cell response has been linked to lethal COVID-19 infection, which underscores the crucial role of these cells (31, 32) to control infection and disease. Acute SARS-CoV-2 infection is characterized by a strong decrease in naïve CD4^+^ T cells, especially in severe cases (33), with an increased frequency of many effector memory cell subsets. PD-1 (Programmed cell death protein 1) upregulation has been found in all subsets of CD4^+^ T cells, with the exception of the naïve compartment, in combination with other exhaustion markers (34, 35), denoting a dysfunctional/exhausted CD4^+^ phenotype in severe cases.

In contrast, CD8^+^ T cells are critical for virus clearance by killing infected cells that present viral antigens by class I MHC (Major histocompatibility complex) molecules. As expected, the expansion of virus-specific CD8^+^ T cells in COVID-19 has been associated with better clinical outcomes (28, 36). Also for CD8^+^ T cells, Spike, followed by M, and Nucleocapsid are the dominant antigens (36–39). Both SARS-CoV-2 specific CD4^+^ and CD8^+^ T cells are already identified in the first few days after symptom onset (40). Peripheral CD8^+^ T cells have cytotoxic function and secrete cytokines (41) with an increased frequency of HLA-DR^+^/CD38^+^ cells, especially in patients progressing to severe disease. At the same time, numerous studies have reported a dysfunctional, exhausted phenotype of the CD8^+^ T cell compartment linked to disease severity (27, 42) and a propensity for apoptosis as indicated by increased levels of TRAIL-receptor (TNF-related apoptosis-inducing ligand receptor) and CASP3 (Caspase 3) (43).

Intriguingly, the characterization of SARS-CoV-2 antigen-specific T cells in the periphery revealed that different epitopes polarize the CD4^+^ T cell response towards distinct outcomes. Anti-spike CD4^+^ T cells mainly show a Tfh phenotype (44) which is contrasted by a Th1/Th17 polarization of M- and Nucleocapsid-specific T cells (44). Interestingly, CD8^+^ T cells specific for M and Nucleocapsid are more polyfunctional than anti-Spike cells, a finding that has not yet sufficiently been considered in vaccine strategies, which currently target only Spike as antigens (29). Of relevance, antigen-specific T cell activation does not seem to be followed by exhaustion during acute infection and convalescence (45).

In mice SARS-CoV-2 induces a robust T cell response where both CD4^+^ and CD8^+^ T cells are important for virus clearance (46, 47). In addition, similarly to humans, the Type I interferon pathway is critical for the generation of robust T cell responses against SARS-CoV-2 infection in the airways of infected mice (47). Even though, none of the animal models could fully recapitulate the severe SARS-CoV-2 phenotype seen in the lung of humans, upregulation of T cell associated and pro-inflammatory cytokines was observed in the lung of SARS-CoV-2 infected K18-hACE2 transgenic mice (expressing the human angiotensin I converting enzyme 2 (ACE2) receptor) (48). In light of the differences between SARS-CoV-2 infection in humans and the current animal models, this review focuses on the reported human phenotypes.

### Protective role of T cells in COVID-19

**Figure 2:**
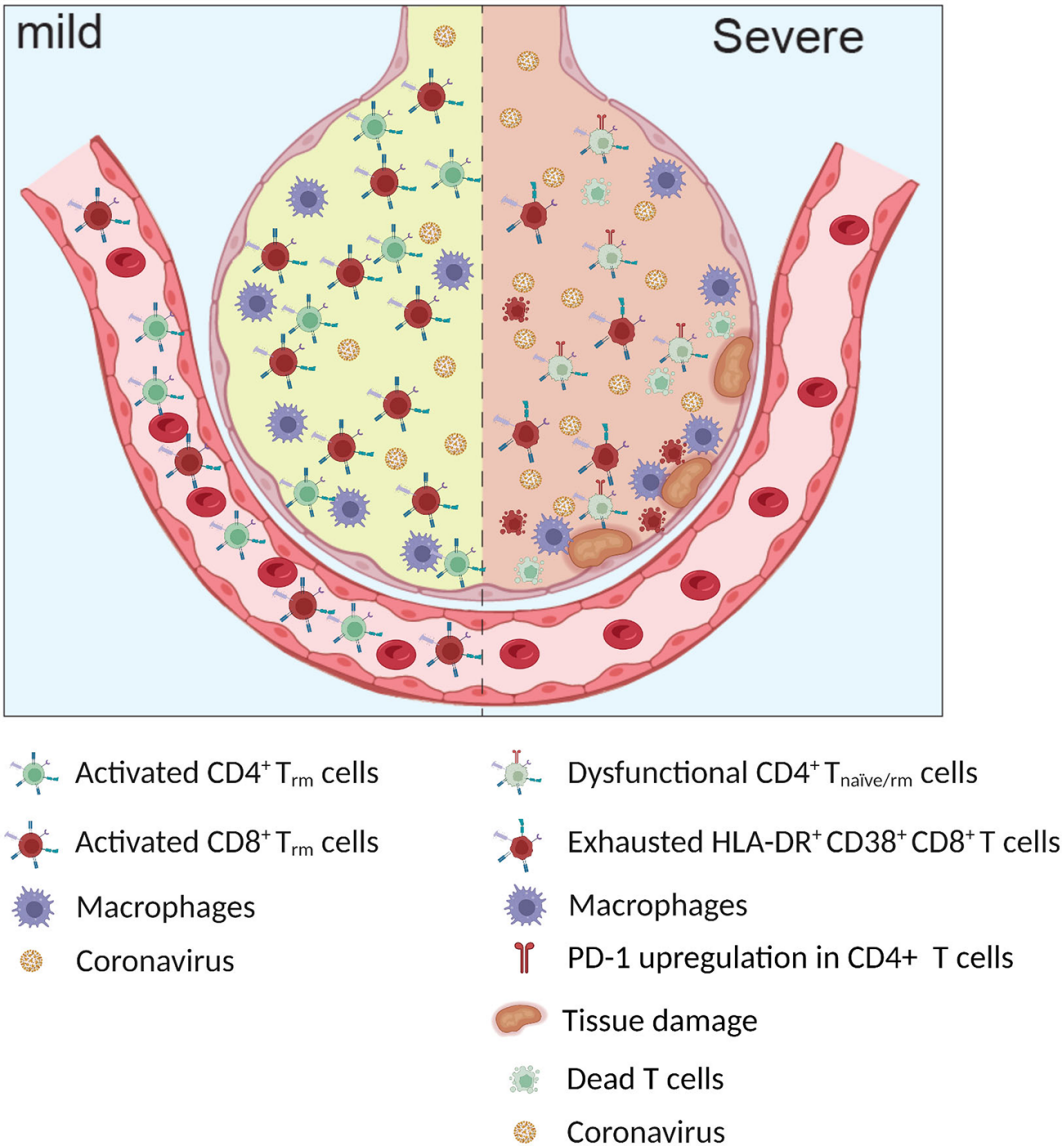
Infected alveoli in mild and severe Covid-19. Higher frequency of exhausted HLA-DR^+^ CD38^+^ CD8^+^ T cells and dysfunctional CD4^+^ T _naïve/rm_ cells observed in patients with severe COVID-19.

Most measurements of human adaptive immunity are performed in circulating cells because the blood is by far the most accessible tissue to be investigated as a proxy for tissue immune responses. With this mini-review, we do not aim to provide a comprehensive summary of peripheral T cell phenotypes; we will focus instead on the T cell response to SARS-CoV-2 in the lungs, as the immunological profiles in the two tissues differ (49) (**Figure 2**).

As peripheral lymphopenia was described early on in the pandemic as a hallmark of COVID-19, which also positively correlated with disease severity, the question was whether these cells migrated to the tissue or suffered activation-induced cell death (AICD). Interestingly, early studies on bronchoalveolar lavage (BAL) found an increased number of infiltrating T cells in moderate rather than severe cases, suggesting a protective role of T cells in the lung. In contrast, severe cases displayed massive AICD in the periphery, resulting in a lack of migration of T cells into their infected tissues (50).

Clinically, the lack of tissue-resident T cells in the lungs of severe COVID-19 patients, together with a lack of expanded SARS-CoV-2 specific T cells in favor of a more dysfunctional/exhausted phenotype, seems to be the most widely confirmed phenotype (32, 50, 51).

Liao and colleagues were among the first to describe the phenotype of lung T cells in COVID-19. Apart from the lower number of CD8^+^ cells, they found a higher frequency of proliferating cells in severe compared to moderate disease (50). These cells also expressed cytotoxic genes (*GZMA*, *GZMK*, and *FASLG*) at high levels. In contrast, genes involved in T cell activation, migration, and cytokine secretion were elevated in moderate cases (50). Investigating the clonality of the T cell response, the authors found that moderate cases had a more robust clonal expansion, as well as enriched expression of tissue residency markers (*XCL1*, *CXCR6*, and *ITGAE*). This suggests a more coordinated T cell response in mild/moderate cases that is able to induce a T_rm_ phenotype potentially beneficial for viral clearance and long-term protection from reinfection (50). These results were also confirmed by an independent report (52), where the authors highlighted that in severe cases, CD4^+^ T cells exhibit a more naïve phenotype (*IL7R*, *CCR7*, *S1PR1*), again pointing towards a dysfunctional T cell responses in severe COVID-19.

While the early reports certainly elucidated important aspects of the pathomechanisms of SARS-CoV-2 infection, the limited number of patients investigated made it challenging to model disease progression over time. In a later study, Wauters and colleagues included more than 30 patients in their single-cell transcriptomic study and inferred activation/differentiation trajectories across cell types and disease severity (51). Here, a more efficient cross-talk between T cells and the lung microenvironment in mild/moderate patients was described, further supporting the notion that the adaptive immune compartment is critical for resolving the disease (51).

In this cohort, the authors identified a moderately increased frequency of MAIT cells (Mucosal-associated invariant T) and a marked increase in T_rm_ cells in moderate compared to severe cases, consistent with previous observations (51). Further trajectory analysis clarified that CD8^+^ T cells from patients with moderate disease courses were enriched in the T_rm_ differentiation trajectory, whereas those from severe patients branched into an exhausted phenotype (51). TCR analysis of the CD8^+^ compartment also confirmed previous findings that mild and moderate patients developed clonally expanded, potentially antigen-specific, T_rm_ populations, supporting a protective role of the antigen-specific T cells in these patients (51).

A similar trajectory analysis in the pulmonary CD4^+^ compartment revealed a strong polarization of helper cells to Th1/Th17 in COVID-19, also denoted by a dysfunctional phenotype (according to gene expression) in severe disease. TCR analysis showed that these cells are enriched for expanded clones most probably specific for SARS-CoV-2 (51).

### The pathological potential of T cell response

Despite a clear association between SARS-CoV-2 specific T cell responses and mild and moderate COVID-19, interpretable as a predominantly protective role of the adaptive response in disease progression (53, 54), other reports have pointed towards a dysfunctional response in severe cases (50, 51). For example, a higher activation state in the overall lymphoid compartment was associated with critical COVID-19 disease (55).

Many reports have indicated an intricate relationship between T cell response and local inflammatory environment, pointing toward a different qualitative, rather than quantitative, response in moderate and severe COVID-19. In this context, several groups have reported a potential pathogenic role of the lung T cell response in COVID-19 contributing to organ damage (45, 56, 57) (**Figure 2**).

Chua and colleagues used single-cell transcriptomics on lung tissue samples to investigate the interaction between immune cells and lung epithelial cells. In line with the potentially pathological role of the T cell response in severe/critical COVID-19, the high immunological interaction between immune cells and epithelium suggested that this intricate interplay could potentially lead to uncontrolled tissue damage (45).

More recent studies addressed whether tissue damage is mediated only by SARS-CoV-2 specific T cells or if other mechanisms are also involved. In a multi-omic study, Bergamaschi and colleagues analyzed a longitudinal cohort of more than 200 patients, where they identified a late-onset and prolonged bystander CD8^+^ T cell response (56). The authors speculated that these cells could migrate to the lung *via* CXCR3, leading to NKG2D-dependent killing of non-infected lung cells (56, 58).

Furthermore, supporting the direct pathological role of T cells in the lung, we recently identified a population of CD16^+^ T cells both in circulation and in the lung parenchyma (57). We showed how these cells are induced by the inflammatory microenvironment *via* complement activation and can adopt antibody-dependent cellular cytotoxicity in response to immune complexes similar to innate immune cells, leading to tissue damage *via* a TCR-independent mechanism (57). The frequency of these cells was also associated with severe disease and was shown to be a strong prognostic marker for disease severity (57).

It is important to acknowledge that all the above-mentioned studies only partially addressed the dynamics of disease progression in the lung due to the clear difficulties in longitudinal sample procurement. The stage at which the protective response diverges into a dysfunctional phenotype is unclear and requires further insights to fully recapitulate the local processes involved.

Taken together, it is established that T cells in the lung have a dual role during COVID-19, with a more coordinated/protective response driving mild/moderate disease and a dysfunctional/tissue-damaging response in severe disease. It is important to note that T cells are not the only immune cell type with a dysfunctional response in severe COVID-19, as especially lung neutrophils (59) and macrophages (60) were shown to contribute to extensive tissue damage and fibrosis.

## Open questions and future perspectives

Research efforts to better understand SARS-CoV-2 infection and COVID-19 disease have been tremendous over the last three years. Studies across the world have shed light on the role of the immune system and in particular on T cells in both the disease course and the protection from infection and severe disease. The unexpectedly high diversity of the disease burden and the heterogeneity of clinical symptoms but also pathological findings were unexpected and are certainly one major reason why still many questions are open. Answering these questions is of particular interest since protecting the general public from severe infections remains a key priority of our health system. Therefore, to further increase our knowledge about T cell function, activation, T cell monitoring, and dynamics of TCR repertoire of SARS-CoV-2 specific T cells after infection or vaccination is crucial. What antigen induces long-term memory and is a type I response always advantageous? Are the clones cross-reactive with other virus mutants? Would another administration route of the vaccine be beneficial to induce a population of antigen-specific T_rm_ cells in the lungs?

Beyond that, the existence of long-term consequences of infection, e.g. long-COVID (61) poses an additional challenge for the health system on a global level. And also here, our understanding of the role of T cells in the pathophysiology of long-COVID is far from understood and heavily discussed (62).

## Closing remarks

As in most viral infections, T cells play a critical role in the response to SARS-CoV-2 and immunological memory formation, protecting against (re)infection after vaccination or previous SARS-CoV-2 exposure. In this process, tissue-resident T_rm_ cells are pivotal, as they can act as safeguards at the main virus entry point. Over the last 3 years, the scientific community greatly benefited from omic approaches to understand the complex pathomechanism behind COVID-19 (63, 64), shedding light on the difference in T cell response between mild/moderate and severe disease. Nevertheless, further studies are required to fully understand the local response to SARS-CoV-2 and to potentially reveal novel opportunities for targeted treatment and vaccination.

## Author contributions

LB drafted the manuscript, MS prepared the figures. LB, IG, MS, and MB revised and finalized the manuscript. All authors contributed to the article and approved the submitted version.
